# Self-consistency and congruence, perceived social support, and psychological resilience as predictors of professional identity of male nursing students among consecutive secondary and higher vocational education

**DOI:** 10.3389/fpsyg.2025.1588734

**Published:** 2025-06-26

**Authors:** Li Ma, Xu Sun, Wei Zhou, Zhenpeng Lin, Anqi Li, Fanqun Kong, Yuchen Xue, Zhouyan Dong, Yulong Wu, Mei Cheng

**Affiliations:** ^1^School of Nursing, Binzhou Medical University, Yantai, China; ^2^Department of Nursing Education, Yantai Nurses School of Shandong, Yantai, China; ^3^Department of Pathogenic Biology, Binzhou Medical University, Yantai, China

**Keywords:** male nursing students, professional identity, psychological resilience, social support, self-consistency and congruence

## Abstract

**Objective:**

Considering the traditional female nature of the nursing field, male nursing students often experience a strong urge to leave the profession. A robust professional identity (PI) is crucial for male nurses, as it can alleviate professional stress and enhance their willingness to remain in the field. Among full-time nursing vocational education programs, the consecutive secondary and higher vocational nursing (CSAHVN) stands out for offering comprehensive and advanced professional education, leading to the cultivation of highly skilled nurses. Nevertheless, the predictive factors influencing male nursing students’ PI during their CSAHVN education remain poorly understood.

**Methods:**

Convenience sampling was used to recruit male students in the CSAHVN education from two vocational schools. Data on PI, self-consistency and congruence, perceived social support and psychological resilience were collected using questionnaires.

**Results:**

The average score for male nursing students’ PI was 3.85 (SD = 0.68). The total scores and sub-dimensions of self-consistency and congruence, perceived social support, and psychological resilience demonstrated significant correlations with PI. Multivariate analyses identified motivation and interest in nursing profession, along with sub-dimensions of self-consistency and congruence (self-flexibility), psychological resilience (tenacity), and perceived social support (family support) (*p* < 0.01) as principal predictors of the PI among male CSAHVN students, with three-dimensional scatter plots demonstrating superior predictive capacity of tenacity and self-flexibility.

**Conclusion:**

Our findings underscore the necessity for systemic interventions in educational innovations that strengthen professional commitment and foster family-school collaboration to enhance tenacity and self-flexibility, which are essential to counteract gender stereotypes and thereby increase the PI of male students in CSAHVN education.

## 1 Introduction

Professional identity (PI) refers to a person’s affirmative evaluation of the occupation, thus forming the desire to adhere to the career choice, career motivation and career loyalty (Fagermoen, 1997). PI plays a vital role in enabling nurses to provide high-quality care, which can reduce the negative impact of work pressure and improve work performance and retention willingness (North et al., 2013; Sabanciogullari and Dogan, 2015). Given the historically female-dominated nature of nursing, male nurses face various cognitive and social biases. Consequently, the PI crisis among male nurses is particularly severe, as they experience more stress and job-related emotions compared to female nurses (Zeabadi et al., 2021). The formation of nurses’ PI begins at the professional education stage (Arreciado Maranon and Isla Pera, 2015; Henry, 1993), where a positive PI is beneficial to improve nursing students’ professional satisfaction, enhance their sense of professional achievement (Browne et al., 2018), and reduce their turnover tendency after engaging in this profession (Chenevert et al., 2016). Herein, identifying the status and influencing factors of male nursing students’ PI is helpful to early interventions of PI for male nursing practitioners.

Vocational educational nursing students constitute the largest part of the nursing workforce. Among the full-time nursing vocational education, the consecutive secondary and higher vocational nursing (CSAHVN) education can provide subsequential and enhanced educational services, consequently producing more skilled nurse capital. Nursing students in this educational model are programmed to pass the secondary vocational education and go directly to the higher vocational education, and will finally take the nursing credentialing and licensure examination for registered nurses (NCLEX-RN) exam, equivalent to an associate degree in the United States. This educational model has become a popular option of nursing vocational education due to its lower admission standards and relatively high educational gains. However, most of the studies on male nursing students’ PI focused on those enrolled in undergraduate education (He et al., 2022; Wang et al., 2011), leaving the current status and influencing factors of PI on male nursing students in the CSAHVN education largely unexplored.

The theoretical model for PI is based on general ego identity models. The formation and development of ego identity are driven by positive feelings of self-determination (Ryan and Deci, 2000b). Based on the self-determination theory, fundamental human psychological needs include competence, relatedness and autonomy (Deci et al., 1994). If individuals are supported by these needs, they will internalize their roles or activities and obtain high-level motivation to promote a positive ego identity (Mylrea et al., 2017). “Competence” means that people can exert an impact on the surrounding environment (Mylrea et al., 2017). “Autonomy” can be considered as authorship or self-congruence, referring to the individual experience of being the actor of one’s behavior (Weinstein et al., 2012). The individual is believed to show a stronger sense of professional mission when the multiple egos are in a self-consistency and congruence state (Hagmaier and Abele, 2015). Psychological resilience refers to individuals’ sense of ability to control and have an impact on their environment successfully (Campbell-Sills and Stein, 2007). An experimental study found that psychological resilience can motivate nursing students to cope successfully with adverse environments (Skinner et al., 2013). “Relatedness” refers to connecting with others (Mylrea et al., 2017). Social support can explicitly enhance social connectivity and a sense of relatedness among individuals. Perceived social support, as a positive experience generated when individuals interact with others, could help nurses improve self-efficacy, reduce work pressure, and prevent them from leaving the profession (Liu and Aungsuroch, 2019; Yu et al., 2021).

In light of the above concerns, we are wondering whether psychological resilience, perceived social support, self-consistency and congruence may influence the PI of the male nursing students enrolled in the CSAHVN education. A multicenter cross-sectional study was conducted to explore these students’ PI and to confirm associations between the sociodemographic characteristics, psychological resilience, perceived social support, self-consistency and congruence, and the PI. The present study will assist nursing educators in developing strategies to increase male nursing students’ PI, which affects their physical and mental health and the quality of care they provide.

## 2 Materials and methods

### 2.1 Research design

The convenience sampling method was used for a multicenter cross-sectional survey in our study. The present research was reported in accordance with the STrengthening the Reporting of OBservational studies in Epidemiology (STROBE) checklist (Elm et al., 2007). This study was approved by Binzhou Medical University’ s Ethic Commission.

### 2.2 Participation and setting

We recruited male nursing students from the consecutive secondary and higher vocational education nursing schools located in Shandong Province, China. Inclusion criteria: (1) Full-time male students enrolled in the “3 + 2” CSAHVN program, whose normal studies were not interrupted at school for more than half a year; (2) Have normal language expression and communication skills; (3) Voluntary participation with signed informed consent. Exclusion criteria: (1) Students with prior healthcare work experience; (2) Interrupted enrollment during data collection.

### 2.3 Measures

The Self-Consistency and Congruence Scale (SCCS) was designed by Wang based on the seven dimensions proposed by Rogers (Wang, 1994). This scale consisted of 35 items which were grouped into three subscales: disharmony of self and experience, self-flexibility and self-stereotype. A higher total SCCS score (the score of the self-flexibility subscale is reversed) indicates a lower level of self-consistency. The reported Cronbach’s α coefficients were 0.85, 0.81, and 0.64, respectively.

The Perceived Social Support Scale (PSSS) (Zimet et al., 1990) uses a 12-item scale that measures individuals’ perceptions of support from family, friends and a significant other. A higher score on the scale indicated a higher perceived social support. The reported internal consistency was 0.88 and test–retest reliability was 0.85.

The Chinese version of the Connor-Davidson Resilience Scale (CD-RISC) was translated and revised by Xiaonan Yu and Jianxin Zhang (Yu et al., 2007). This scale consisted of 25 items and was grouped into three subscales: tenacity, strength, and optimism. Higher scores indicated a higher resilience. The reported Cronbach’s α coefficients varied from 0.86 to 0.94.

The professional identity questionnaire for nursing students (PIQNS) was designed in the Chinese language (Mandarin) to measure nursing students’ PI (Hao, 2011). The scale contains 17 items that are distributed across the following five dimensions: professional self-concept, retention benefit and risk of turnover, social comparison and self-reflection, career choice dependence, and social modeling. A higher scale indicates better PI. The reported Cronbach’s α coefficients varied from 0.86 to 0.94.

Other variables included participants’ demographic data such as age, birthplace, grade, whether the family is a single-child family, household composition, monthly income, and reasons for choosing nursing vocational education.

### 2.4 Data collection

Data collection was conducted for male students between March–June 2024. After soliciting the informed consent, the questionnaires were subsequently distributed to those who signified their voluntary participation. It took approximately 10–15 min to complete the questionnaire. The staff were responsible for answering any questions in the process of filling out the questionnaire, and the staff were present during the whole process of filling out the questionnaire. After recovering the questionnaires, we entered the data into Excel with a double check.

### 2.5 Data analysis

The statistical software SPSS 26.0 was employed to perform descriptive statistics, Pearson correlation analysis, and multiple linear regression analysis. Student *t*-test and ANOVA were employed to test the differences of each demographic characteristic on PI scores. Post-hoc comparisons were conducted using the Bonferroni *post-hoc* test. Pearson correlation analysis was adopted to explore the relationship among self-consistency and congruence, perceived social support, psychological resilience, and PI. Multiple liner regression analyses were employed to explore the factors influencing the total PI scores. Analyses were conducted using Python version 3.6 (Python Software Foundation). Data for the independent variables were prepared as NumPy arrays, and the model was fitted using the LinearRegression class from the sklearn.linear_model module. Predictions were generated and visualized in a 3D scatter plot using Matplotlib. The statistical significance was set at a *p*-value < 0.05.

## 3 Results

### 3.1 Sociodemographic characteristics of male nursing students and variations in their PI

A total of 394 questionnaires were received from participants. Out of these, 335 questionnaires were deemed valid, resulting in an effective recovery rate of 85.03%. Table 1 listed the personal characteristics of the participants with an average age of 17.54 ± 1.58. Levene’s test indicated homogeneity of variance across all groups (*p* > 0.05), validating the assumptions for subsequent ANOVA analyses. The results demonstrating that the scores of PI did not differ by birthplace, whether they were the only child and monthly income. By contrast, significant differences were observed in male nursing students’ PI across grade, household composition, and the reason for choosing nursing vocational education (*p* < 0.01).

**Table 1:** Characteristics of male nursing students and univariate analysis of the factors related to their PI (*n* = 335).

Variable	Categories	Number of participators *n* (%)	Mean ± SD	*t/F*	*p*
Birthplace	Rural	171 (51.04)	65.23 ± 11.30	0.001	0.656
	Urban	164 (48.96)	65.80 ± 11.86		
From single-child family	Yes	236 (70.45)	66.21 ± 11.37	0.264	0.091
	No	99 (29.55)	63.82 ± 11.93		
Grade	Grade 1	73 (21.79)	67.67 ± 10.71	9.411	*p* < 0.001
	Grade 2	72 (21.49)	62.51 ± 10.62		
	Grade 3	71 (21.19)	68.07 ± 12.10		
	Grade 4	70 (20.90)	60.26 ± 10.10		
	Grade 5	49 (14.63)	70.45 ± 11.69		
Household composition	Dual-parent household	288 (85.97)	66.22 ± 11.48	*p* < 0.001	0.005
	Other (single-parent/orphan)	47 (14.03)	61.11 ± 11.28		
Monthly income (RMB)	<2,000	13 (3.88)	58.46 ± 16.43	2.692	0.069
	2,000 ~ 6,000	195 (58.31)	66.08 ± 11.08		
	>6,000	127 (37.91)	65.34 ± 11.60		
Reasons for choosing nursing vocational education	Motivation and interest in nursing profession	145 (43.28)	70.44 ± 11.23	21.689	*p* < 0.001
	Parents’ wishes	130 (38.81)	61.48 ± 10.89		
	Recommendations of others	60 (17.91)	62.28 ± 9.20		

SD, standard deviation.

### 3.2 Scores of PIQNS, SCCS, PSSS, and CD-RISC among male nursing students enrolled in the CSAHVN education

The total scores of PIQNS, SCCS, PSSS, and CD-RISC were 65.50 ± 11.57, 92.64 ± 14.87, 67.16 ± 12.33 and 97.46 ± 15.21, respectively (Table 2).

**Table 2:** Mean scores (Min, Max, Mean ± SD) of the PIQNS, SCCS, PSSS, and CD-RISC for male students (*n* = 335).

Variables	Sub-dimensions	Min	Max	Mean ± SD
PIQNS	Professional self-image	1.00	5.00	3.92 ± 0.79
	Retention benefit and risk of turnover	1.00	5.00	3.60 ± 0.85
	Social comparison and self-reflection	1.00	5.00	3.99 ± 0.73
	Career choice dependence	1.50	5.00	3.79 ± 0.79
	Social modeling	1.00	5.00	4.00 ± 0.93
	Overall mean score	1.24	5.00	3.85 ± 0.68
SCCS	Disharmony of self and experience	1.00	5.00	2.97 ± 0.70
	Self-flexibility	1.00	4.42	2.08 ± 0.51
	Self-stereotype	1.00	5.00	2.88 ± 0.69
	Overall mean score	1.40	3.63	2.65 ± 0.42
PSSS	Family support	1.00	7.00	5.63 ± 1.20
	Friends support	1.50	7.00	5.54 ± 1.17
	Others support	2.25	7.00	5.60 ± 1.09
	Overall mean score	2.17	7.00	5.60 ± 1.03
CD-RISC	Tenacity	1.23	5.00	3.85 ± 0.66
	Strength	1.13	5.00	4.15 ± 0.61
	Optimism	1.50	5.00	3.56 ± 0.70
	Overall mean score	1.24	5.00	3.90 ± 0.61

PIQNS, Professional Identity Questionnaire for Nursing Students; SCCS, Self-Consistency and Congruence Scale; PSSS, Perceived Social Support Scale; CD-RISC, Connor-Davidson Resilience Scale; SD, standard deviation.

The overall mean score for the PIQNS (on a 5-point scale) was 3.85 (SD = 0.68), with the social modeling dimension ranking first by a weak advantage (mean value = 4.00, SD = 0.93). The scores of retention benefit and risk of turnover dimensions were the lowest (mean value = 3.60, SD = 0.85) (Table 2).

The overall mean score for SCCS (on a 5-point scale) was 2.65 (SD = 0.42), with the highest score of disharmony of self and experience dimension (mean value = 2.97, SD = 0.70), and the lowest score of self-flexibility dimension (mean value = 2.08, SD = 0.51) (Table 2).

The overall mean score for PSSS (on a 7-point scale) was 5.60 (SD = 1.03), with the highest score of family support dimension (mean value = 5.63, SD = 1.20) and the lowest score of friends support dimension (mean value = 5.54, SD = 1.17) (Table 2).

The overall mean score for CD-RISC (on a 5-point scale) was 3.90 (SD = 0.61), with the highest score of the strength dimension (mean value = 4.15, SD = 0.61), and the lowest score of the optimism dimension (mean value = 3.56, SD = 0.70) (Table 2).

### 3.3 Correlations of the total scores and sub-dimensions of PIQNS, SCCS, PSSS, and CD-RISC among the male nursing students among the male nursing students enrolled in the CSAHVN education

**Figure 1:**
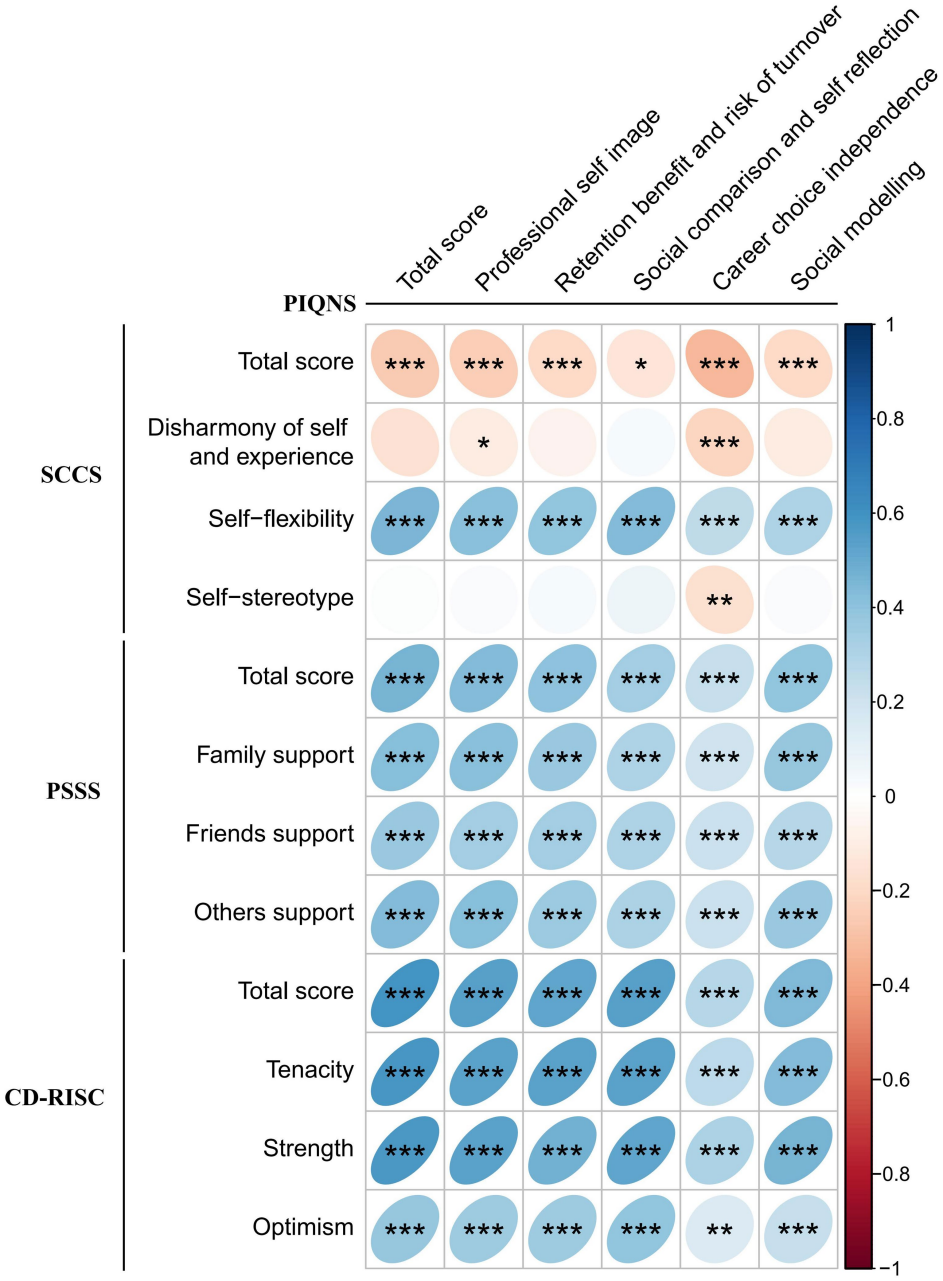
Correlation diagram of the total scores and sub-dimensions of PIQNS, SCCS, PSSS, and CD-RISC of male nursing students (*n* = 335). PIQNS, Professional Identity Questionnaire for Nursing Students; SCCS, Self Consistency and Congruence Scale; PSSS, Perceived Social Support Scale; CD-RISC, Connor-Davidson Resilience Scale; SD, standard deviation. *p < 0.05, **p < 0.01, ***p < 0.001.

Table 3 and Figure 1 indicated that the overall scores and sub-dimensions of the PIQNS for male students exhibited a significant negative correlation with the total scores of the SCCS and its self-flexibility dimension (*p* < 0.01). In contrast, these scores were positively correlated with the total scores and each sub-dimension of the PASS and the CD-RISC (*p* < 0.01).

**Table 3:** Correlations of the total scores and sub-dimensions of PIQNS, SCCS, PSSS, and CD-RISC of male nursing students (*n* = 335).

	PIQNS					
	Total score	Professional self-image	Retention benefit and risk of turnover	Social comparison and self-reflection	Career choice independence	Social modeling
SCCS						
Total score	−0.259**	−0.243**	−0.199**	−0.132*	−0.323**	−0.194**
Disharmony of self and experience	−0.106	−0.110*	−0.066	0.030	−0.218**	−0.103
Self-flexibility	0.452**	0.413**	0.392**	0.438**	0.258**	0.301**
Self-stereotype	0.016	0.026	0.035	0.077	−0.165**	0.020
PSSS						
Total score	0.461**	0.439**	0.405**	0.348**	0.237**	0.390**
Family support	0.427**	0.412**	0.371**	0.309**	0.204**	0.380**
Friends support	0.375**	0.340**	0.344**	0.304**	0.213**	0.288**
Others support	0.430**	0.421**	0.366**	0.315**	0.216**	0.374**
CD-RISC						
Total score	0.590**	0.542**	0.520**	0.545**	0.283**	0.443**
Tenacity	0.586**	0.537**	0.531**	0.533**	0.269**	0.439**
Strength	0.578**	0.532**	0.477**	0.528**	0.316**	0.467**
Optimism	0.385**	0.358**	0.352**	0.393**	0.158**	0.238**

*p < 0.05, **p < 0.01.

PIQNS, Professional Identity Questionnaire for Nursing Students; SCCS, Self Consistency and Congruence Scale; PSSS, Perceived Social Support Scale; CD-RISC, Connor-Davidson Resilience Scale; SD, standard deviation.

### 3.4 Multivariate predictors and dimensional visualization of the PI among male nursing students enrolled in the CSAHVN education

**Figure 2:**
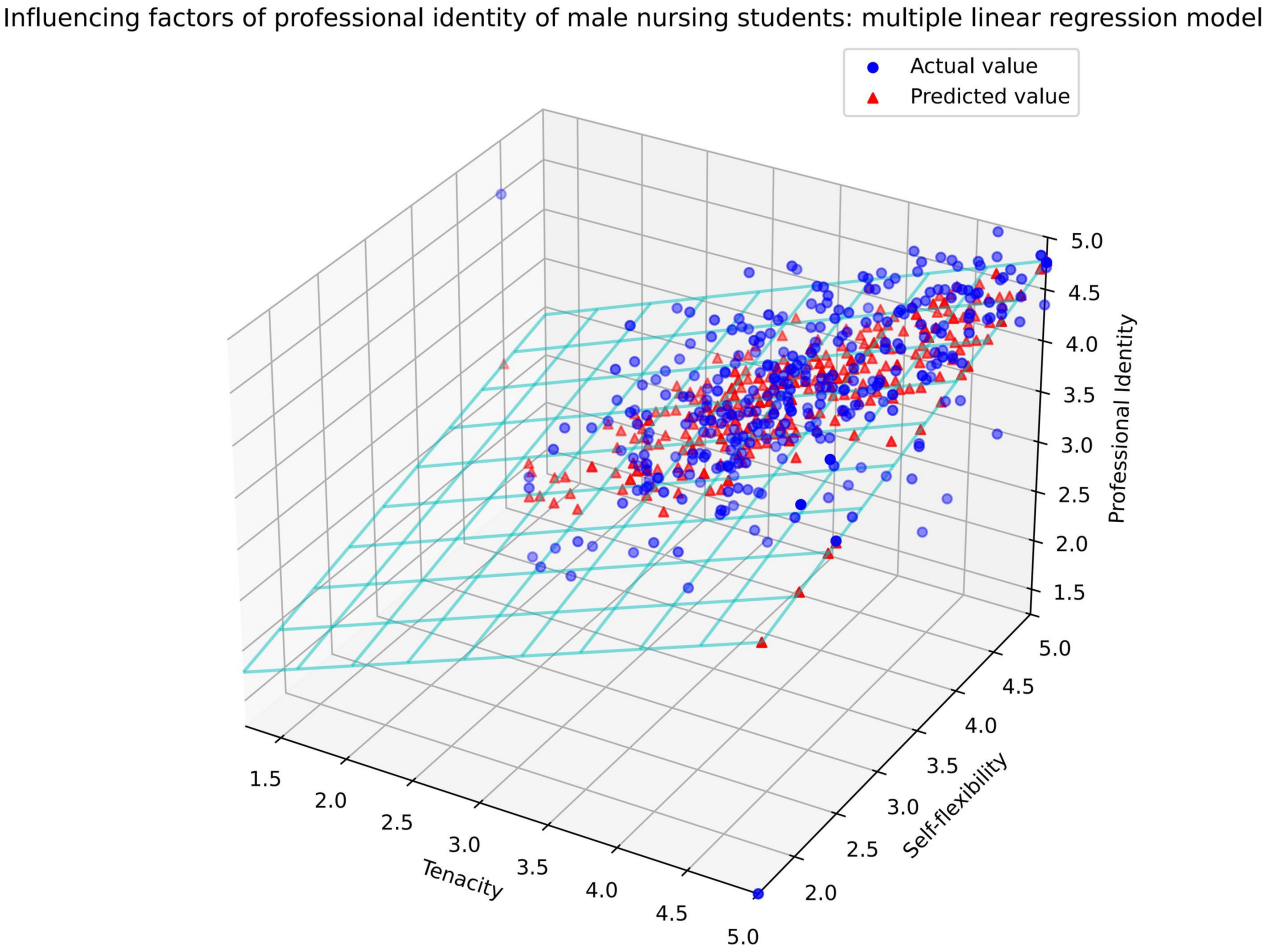
3D scatter plot of tenacity and self-flexibility predicting professional identity. The axes represent the scores of Tenacity (X-axis) and Self-Flexibility (Y-axis), while the Z-axis represents the predicted scores of PI. The scatter plot displays actual values (blue dots) and predicted values (red triangles) of PI. The wireframe surface illustrating the regression plane. PI: Professional Identity.

The multiple liner regression analysis (Table 4) identified that motivation and interest in nursing profession, sub-dimensions of self-consistency and congruence (self-flexibility), psychological resilience (tenacity), and perceived social support (family support) as principal predictors of PI in male CSAHVN students (*F* = 56.371, *R*^2^ = 0.461, adjusted *R*^2^ = 0.453, *p* < 0.001). Three-dimensional scatter plots (Figure 2) demonstrated superior predictive capacity of tenacity and self-flexibility on the male students’ PI. Our findings underscore the need to implement well-structured educational initiatives to enhance motivation and interest in nursing education. Of particular significance is the development of bidirectional home-school partnerships that incorporate interventions aimed at fostering tenacity and self-flexibility, which can assist male students in coping with the stressors associated with the perception of nursing as a female-dominated profession.

**Table 4:** Influencing factors of PI of male nursing students: multiple linear regression model (*n* = 335).

Variables	*R* ^2^	*B*	*SE*	β	*t*	*p*
Constant		0.670	0.233		2.878	0.004
Tenacity	0.343	0.400	0.049	0.390	8.121	*p* < 0.001
Self-flexibility	0.204	0.276	0.061	0.206	4.560	*p* < 0.001
Reasons for choosing nursing vocational education	0.139					
Motivation and interest in nursing profession Parents’ wishes Recommendations of others^ref.:^		0.303	0.058	0.221	5.248	*p* < 0.001
Family support	0.182	0.076	0.026	0.134	2.907	0.004

B is unstandardized coefficient; SE is standard error; β is standardized coefficient; ref. = reference.

## 4 Discussion

The male secondary vocational or higher vocational nursing school students are a vital and indispensable backup force for the nursing profession. Among the full-time vocational nursing education, the CSAHVN education can provide subsequential and enhanced vocational education for nursing students. This study investigated the current status and influencing factors of the PI in male CSAHVN students. The findings of this study indicated that motivation and interest in nursing profession, along with sub-dimensions of self-consistency and congruence (self-flexibility), perceived social support (family support), and psychological resilience (tenacity) emerged as the primary predictors of the PI among male nursing students enrolled in the CSAHVN education programs. To our knowledge, this study pioneers the integration of self-consistency with resilience and social support frameworks to elucidate the multifaceted predictors of PI in male nursing students within the CSAHVN education. Our findings underscore the significance of incorporating targeted interventions, such as bolstering career motivation workshops, promoting family engagement programs, and introducing resilience-building training into CSAHVN curricula to reinforce the professional identity of male nursing students. This approach aims to tackle workforce shortages and enhance gender diversity within clinical nursing practice.

In this study, the PI of male CSAHVN students was at a moderate level, similar to the PI of the male nursing students in the 3-year junior college (Chen et al., 2020) and the first-year post-associate degree baccalaureate nursing students in China (Wu et al., 2020), which warrants immediate attention. However, the PI of male CSAHVN students was a little higher than that of the Chinese male undergraduate nursing students (Qiu et al., 2020; Tang et al., 2020; Zhou et al., 2021). In China, nurses with different education degrees perform similar duties and have similar salaries with approximately $50 monthly income difference in most hospitals (Chen et al., 2020). This mismatch between educational effort and salary income may frustrate the PI of nursing students with bachelor’s degrees and higher, as they have higher expectations for promotion, social status, income, and personal achievement (Guo et al., 2018). Additionally, we found that the score of the male CSAHVN students’ PI displayed a waxing and waning trend with grades, with lower scores in grades two and four and relatively higher scores in grades three and five. These results were inconsistent with previous researchers who reported that the PI of male nursing students in the 3-year junior college and the undergraduate college increased with grades (Chen et al., 2020; Dan et al., 2016). The most possible reason may be that the male CSAHVN students in China are programmed to experience two internship stages in grade three (8 months) and grade five (6 months), respectively. Positive interpersonal relationships in the clinical learning environment facilitated feelings of acceptance and a sense of belonging to the career workshop, which is important for the development of students’ PI (Sun et al., 2016; Wenrich et al., 2013).

Self-determination theory suggests that intrinsic motivation is the main driving force for outstanding performance (Ryan and Deci, 2000a). Professional interest as an intrinsic motivation was one of the most influential factors affecting career choice and professional adaptation among nursing students (Halperin and Mashiach-Eizenberg, 2014). In our study, we found that the motivation and interest in nursing profession is a major predictor of the PI among male CSAHVN students. A survey of Chinese undergraduate nursing students revealed that male students had poor academic performance due to a lack of motivation and interest in learning, which has become an important factor affecting the recruitment and retention of male nursing students (Wang et al., 2011). Students’ decision-making about professional motivation and interests typically begins before matriculation and evolves during their professional studies (Arreciado Maranon and Isla Pera, 2015; Johnson et al., 2012). Hence, it is recommended that nursing educators assess the male nursing students’ professional motivation and interest at enrollment, and provide well-organized educational programs to strengthen their professional motivation and interest during vocational education. Additionally, the government was suggested to broaden the channels to publicize the characteristics and advantages of men in the nursing profession, especially to male adolescents.

It is usually observed that nursing students experience stress from the beginning of their professional education (Turner and McCarthy, 2017). Male nursing students, particularly in settings where male nurses is marginalized by a dominantly female-centric nursing workforce, are found to have excessive stress (Brown, 2009; Wang et al., 2011). Psychological resilience is often defined as an ability to adapt to stress (Connor and Davidson, 2003). A review study in 2019 demonstrated that psychological resilience could improve nurses’ job engagement, job satisfaction, and stay intention, thus creating a well-being nursing team (Yu et al., 2019). Psychological resilience was critical for Chinese nursing students to adjust to mental stress during the novel coronavirus disease (COVID-19), and helped them to develop growing recognition and appreciation of the nursing profession (Zhang et al., 2021). In the present research, we found that the tenacity dimension of psychological resilience is an effective predictor of the male CSAHVN students’ PI. It is recommended that nursing educators should pay attention to gender-related stress and incorporate psychological resilience (especially focus on tenacity) enhancement measures into the vocational education program to smooth the adaptation of male CSAHVN students to professional nursing roles.

Self-consistency and congruence refers to the internal coordination and coordination between oneself and experience (Rogers, 1953), which is a vitalizing, growth-promoting experience that promotes individual’s subjective well-being (Weinstein et al., 2012). Teachers, engineers, and nurses showed a strong professional mission when their multiple internal egos reached self-consistency and congruence (Hagmaier and Abele, 2015). In our research, we found the self-consistency and congruence of the male CSAHVN students were relatively lower than the male teenagers and male college students (Qiu et al., 2020; Tang et al., 2020; Zhou et al., 2021). When men were engaged in the nursing profession, they were prone to encounter gender-specific stereotypes, creating the dilemma of ego identity and eventually leading to inconsistency between oneself and experience (Brown, 2009). Although the male nurses wanted to provide care for the patients, they still felt embarrassed in practice (Zhang and Liu, 2016). In this study, we discovered that the dimension of self-flexibility was incorporated into the regression equation, illustrating it was a predictor of the PI of male CSAHVN students. Self-flexibility refers to the ability to deal with ordinary and unexpected conditions and situational demands (Wang, 1994). Previous researchers suggested that encouraging the recognition of self-worth and improving mental flexibility could efficiently promote the nurses’ professional well-being (van der Riet et al., 2018; Wang et al., 2022). As hospital services have evolved recently, hospitals and society favor male nurses due to physical fitness, stress resistance, and resilience. Thus, we recommend that nursing educators develop specific measures to avoid gender-based stereotypes, improve their awareness of gender predominance, and boost the self-flexibility of male CSAHVN students, consequently enhancing their PI.

PI, a type of social identity, is reported influenced by social support (Huang et al., 2022; Zhao et al., 2023). Social support can be divided into actual social support and perceived social support (Zimet et al., 1990). Compared with actual social support, perceived social support such as experienced and emotional support had more positive effects on the mental health of freshmen (Brissette et al., 2002). In the present study, the family support dimension of perceived social support effectively predicted the PI among male CSAHVN students. Price reported that support from family, friends, and interaction with supervisors could enhance nurses’ job satisfaction and career development aspirations (Price, 2001). Several empirical studies revealed that males chose to engage in the nursing profession because of their family members or acquaintances who have worked as nurses (Harding et al., 2018; Whitford et al., 2020). Among family support, parental support has been shown to influence adolescent mental health, and is deemed as the most effective protective factor against adverse emotions such as depression (Gariepy et al., 2016). Adolescents with high parental support were conducive to facilitating their PI (Dietrich and Salmela-Aro, 2013). Unexpectedly, in our study, we found that the score of family support of male CSAHVN students who chosed to enroll in nursing education by their parents’ wishes were lower than the others (5.29 ± 1.31 vs. 5.86 ± 1.07, *p* < 0.001). The possible reason may be that parents influenced career interests at the admission time, but did not provide ongoing support during vocational education. Williams et al. found that students who perceived patients’ autonomy support, showed fewer health-compromising behaviors (use of alcohol, tobacco, and marihuana) than those who perceived a controlling parental supportive style (Williams et al., 2010). Thereafter, nursing educators should strengthen the home-school cooperation to promote continuous autonomous support from the parents of male CSAHVN students and subsequently foster their PI.

This study is subject to several limitations. The cross-sectional study design excluded the assessment of the timeliness and causality of the observed relationships. In addition, the data relied on self-reported information provided by students, which may possess some potential reporting bias.

## 5 Conclusion

This study found that motivation and interest in nursing profession, along with sub-dimensions of self-consistency and congruence (self-flexibility), perceived social support (family support), and psychological resilience (tenacity) emerged as the primary predictors of the PI among male nursing students enrolled in the CSAHVN education programs. Our findings highlight the importance of providing well-organized educational programs to increase the motivation and interest in nursing education, as well as developing effective home-school collaboration interventions to enhance tenacity and self-flexibility. These strategies help male students cope with stresses associated with stereotypes of the nursing profession as female-centered, thereby promoting the PI of male CSAHVN students and contributing to a stable nursing workforce. To operationalize these insights, we propose three systemic interventions: (1) multidimensional curriculum reform integrating family-involved resilience training and gender-inclusive modules co-developed by vocational education institutions and nursing associations, utilizing clinical simulations and male mentorship to deconstruct stereotypes; (2) aligning government scholarship allocations with regional nursing shortages to incentivize male CSAHVN graduates’ retention in underserved areas; and (3) launching national public health campaigns led by the National Health Commission, leveraging documentaries and social media to rebrand nursing as a gender-neutral profession by showcasing the expertise of male nurses in emergency and critical care.

## Data Availability

The original contributions presented in the study are included in the article/supplementary material, further inquiries can be directed to the corresponding author.

## References

[ref1] Arreciado Maranon A. Isla Pera M. P. (2015). Theory and practice in the construction of professional identity in nursing students: a qualitative study. Nurse Educ. Today 35, 859–863. doi: 10.1016/j.nedt.2015.03.014, PMID: 25863650

[ref2] Brissette I. Scheier M. F. Carver C. S. (2002). The role of optimism in social network development, coping, and psychological adjustment during a life transition. J. Pers. Soc. Psychol. 82, 102–111. doi: 10.1037/0022-3514.82.1.102, PMID: 11811628

[ref3] Brown B. (2009). Men in nursing: re-evaluating masculinities, re-evaluating gender. Contemp. Nurse 33, 120–129. doi: 10.5172/conu.2009.33.2.120, PMID: 19929157

[ref4] Browne C. Wall P. Batt S. Bennett R. (2018). Understanding perceptions of nursing professional identity in students entering an Australian undergraduate nursing degree. Nurse Educ. Pract. 32, 90–96. doi: 10.1016/j.nepr.2018.07.006, PMID: 30098517

[ref5] Campbell-Sills L. Stein M. B. (2007). Psychometric analysis and refinement of the Connor-davidson resilience scale (CD-RISC): validation of a 10-item measure of resilience. J. Trauma. Stress. 20, 1019–1028. doi: 10.1002/jts.20271, PMID: 18157881

[ref6] Chen Y. Zhang Y. Jin R. (2020). Professional identity of male nursing students in 3-year colleges and junior male nurses in China. Am. J. Mens Health 14:1557988320936583. doi: 10.1177/1557988320936583, PMID: 32703068 PMC7383711

[ref7] Chenevert D. Jourdain G. Vandenberghe C. (2016). The role of high-involvement work practices and professional self-image in nursing recruits' turnover: a three-year prospective study. Int. J. Nurs. Stud. 53, 73–84. doi: 10.1016/j.ijnurstu.2015.09.005, PMID: 26421911

[ref8] Connor K. M. Davidson J. R. (2003). Development of a new resilience scale: the Connor-Davidson resilience scale (CD-RISC). Depress. Anxiety 18, 76–82. doi: 10.1002/da.10113, PMID: 12964174

[ref9] Dan C. Weiwei P. Feng G. (2016). Correlation between self-esteem, stress and professional identity of male nursing students in the school. Chin. Nurs. Res. 30, 430–433. doi: 10.3969/j.issn.1009-6493.2016.04.014

[ref10] Deci E. L. Eghrari H. Patrick B. C. Leone D. R. (1994). Facilitating internalization: the self-determination theory perspective. J. Pers. 62, 119–142. doi: 10.1111/j.1467-6494.1994.tb00797.x, PMID: 8169757

[ref11] Dietrich J. Salmela-Aro K. (2013). Parental involvement and adolescents' career goal pursuit during the post-school transition. J. Adolesc. 36, 121–128. doi: 10.1016/j.adolescence.2012.10.009, PMID: 23190942

[ref12] Elm V. Altman D. G. Egger M. Pocock S. J. Gøtzsche P. C. Vandenbroucke J. P.. (2007). The Strengthening the reporting of Observational studies in epidemiology(STROBE) statement: guidelines for reporting Observational studies. BMJ 335, 806–808. doi: 10.1136/bmj.39335.541782.AD 17947786 PMC2034723

[ref13] Fagermoen M. S. (1997). Professional identity: values embedded in meaningful nursing practice. J. Adv. Nurs. 25, 434–441. doi: 10.1046/j.1365-2648.1997.1997025434.x, PMID: 9080267

[ref14] Gariepy G. Honkaniemi H. Quesnel-Vallee A. (2016). Social support and protection from depression: systematic review of current findings in Western countries. Br. J. Psychiatry 209, 284–293. doi: 10.1192/bjp.bp.115.169094, PMID: 27445355

[ref15] Guo Y. J. Yang L. Ji H. X. Zhao Q. (2018). Caring characters and professional identity among graduate nursing students in China-a cross sectional study. Nurse Educ. Today 65, 150–155. doi: 10.1016/j.nedt.2018.02.039, PMID: 29579567

[ref16] Hagmaier T. Abele A. E. (2015). When reality meets ideal: investigating the relation between calling and life satisfaction. J. Career Assess. 23, 367–382. doi: 10.1177/1069072714547164

[ref17] Halperin O. Mashiach-Eizenberg M. (2014). Becoming a nurse - a study of career choice and professional adaptation among Israeli Jewish and Arab nursing students: a quantitative research study. Nurse Educ. Today 34, 1330–1334. doi: 10.1016/j.nedt.2013.10.001, PMID: 24269141

[ref18] Hao Y. F. (2011). Study of the model of self-education in enhancing the level of professional identity and professional self-efficacy in nurse students: Ph. D, The Second Military Medical University.

[ref19] Harding T. Jamieson I. Withington J. Hudson D. Dixon A. (2018). Attracting men to nursing: is graduate entry an answer? Nurse Educ. Pract. 28, 257–263. doi: 10.1016/j.nepr.2017.07.003, PMID: 28739357

[ref20] He X. D. Da Z. Zhang T. Labasabgzhu Zhao J. (2022). Qualitative study on the professional identity of male nursing undergraduates in Tibet universities. Nurs. Pract. Res. 19, 1088–1091. doi: 10.3969/j.issn.1672-9676.2022.07.034

[ref21] Henry P. (1993). Effectiveness of career-development courses for nontraditional premedical students: improving professional identity. Psychol. Rep. 73, 915–920. doi: 10.2466/pr0.1993.73.3.915, PMID: 8302994

[ref22] Huang J. Qiao T. Song Z. Yan J. (2022). How does the social support influence junior college students' occupational identity in pre-school education? Front. Psychol. 13:884606. doi: 10.3389/fpsyg.2022.884606, PMID: 35846679 PMC9280683

[ref23] Johnson M. Cowin L. S. Wilson I. Young H. (2012). Professional identity and nursing: contemporary theoretical developments and future research challenges. Int. Nurs. Rev. 59, 562–569. doi: 10.1111/j.1466-7657.2012.01013.x, PMID: 23134142

[ref24] Liu Y. Aungsuroch Y. (2019). Work stress, perceived social support, self-efficacy and burnout among Chinese registered nurses. J. Nurs. Manag. 27, 1445–1453. doi: 10.1111/jonm.12828, PMID: 31306524

[ref25] Mylrea M. F. Sen Gupta T. Glass B. D. (2017). Developing professional identity in undergraduate pharmacy students: a role for self-determination theory. Pharmacy 5:16. doi: 10.3390/pharmacy5020016, PMID: 28970428 PMC5597141

[ref26] North N. Leung W. Ashton T. Rasmussen E. Hughes F. Finlayson M. (2013). Nurse turnover in New Zealand: costs and relationships with staffing practises and patient outcomes. J. Nurs. Manag. 21, 419–428. doi: 10.1111/j.1365-2834.2012.01371.x, PMID: 23405958

[ref27] Price J. L. (2001). Reflections on the determinants of voluntary turnover. Int. J. Manpow. 22, 600–624. doi: 10.1108/EUM0000000006233

[ref28] Qiu S. L. Liu L. Liu Y. W. Han F. F. Jiang M. M. Mu Y. P.. (2020). Analysis of the current situation of male nursing students' professional identity and influencing factors in a university in tai'an. J. Qilu Nurs. 26, 35–37. doi: 10.3969/j.issn.1006-7256.2020.02.010

[ref29] Rogers C. R. (1953). A research program in client-centered therapy. Res. Publ. Assoc. Res. Nerv. Ment. Dis. 31, 106–118. 13038092

[ref30] Ryan R. M. Deci E. L. (2000a). Intrinsic and extrinsic motivations: classic definitions and new directions. Contemp. Educ. Psychol. 25, 54–67. doi: 10.1006/ceps.1999.1020, PMID: 10620381

[ref31] Ryan R. M. Deci E. L. (2000b). Self-determination theory and the facilitation of intrinsic motivation, social development, and well-being. Am. Psychol. 55, 68–78. doi: 10.1037/0003-066X.55.1.68, PMID: 11392867

[ref32] Sabanciogullari S. Dogan S. (2015). Relationship between job satisfaction, professional identity and intention to leave the profession among nurses in Turkey. J. Nurs. Manag. 23, 1076–1085. doi: 10.1111/jonm.12256, PMID: 25302666

[ref33] Skinner E. Pitzer J. Steele J. (2013). Coping as part of motivational resilience in school: a multidimensional measure of families, allocations, and profiles of academic coping. Educ. Psychol. Meas. 73, 803–835. doi: 10.1177/0013164413485241

[ref34] Sun L. Gao Y. Yang J. Zang X. Y. Wang Y. G. (2016). The impact of professional identity on role stress in nursing students: a cross-sectional study. Int. J. Nurs. Stud. 63, 1–8. doi: 10.1016/j.ijnurstu.2016.08.010, PMID: 27565423

[ref35] Tang L. Jia L. B. Cai Y. Nie Z. K. Li W. L. (2020). Intervention of career planning guidance on male nursing undergraduates by male nurses. J. Nurs. Adm. 20, 644–647. doi: 10.3969/j.issn.1671-315x.2020.09.008

[ref36] Turner K. McCarthy V. L. (2017). Stress and anxiety among nursing students: a review of intervention strategies in literature between 2009 and 2015. Nurse Educ. Pract. 22, 21–29. doi: 10.1016/j.nepr.2016.11.002, PMID: 27889624

[ref37] van der Riet P. Levett-Jones T. Aquino-Russell C. (2018). The effectiveness of mindfulness meditation for nurses and nursing students: an integrated literature review. Nurse Educ. Today 65, 201–211. doi: 10.1016/j.nedt.2018.03.018, PMID: 29602138

[ref38] Wang D. F. (1994). The formation of self-consistency and congruence scale. Chin. J. Clin. Psychol. 2, 19–22.

[ref39] Wang H. Li X. Hu X. Chen H. Gao Y. Zhao H.. (2011). Perceptions of nursing profession and learning experiences of male students in baccalaureate nursing program in Changsha, China. Nurse Educ. Today 31, 36–42. doi: 10.1016/j.nedt.2010.03.011, PMID: 20392548

[ref40] Wang L. Li H. Li X. Zhang J. Lv Y. Jia P.. (2022). Current occupational well-being status and protective and risk factors of male nurses in Chengdu, China: a cross-sectional study. Nurs. Open 9, 1700–1708. doi: 10.1002/nop2.1194, PMID: 35170257 PMC8994956

[ref41] Weinstein N. Przybylski A. K. Ryan R. M. (2012). The index of autonomous functioning: development of a scale of human autonomy - sciencedirect. J. Res. Pers. 46, 397–413. doi: 10.1016/j.jrp.2012.03.007

[ref42] Wenrich M. D. Jackson M. B. Wolfhagen I. Ramsey P. G. Scherpbier A. J. (2013). What are the benefits of early patient contact? A comparison of three preclinical patient contact settings. BMC Med. Educ. 13:80. doi: 10.1186/1472-6920-13-80, PMID: 23731514 PMC3674974

[ref43] Whitford H. M. Marland G. R. Carson M. N. Bain H. Eccles J. Lee J.. (2020). An exploration of the influences on under-representation of male pre-registration nursing students. Nurse Educ. Today 84:104234. doi: 10.1016/j.nedt.2019.104234, PMID: 31707252

[ref44] Williams G. C. Hedberg V. A. Cox E. M. Deci E. L. (2010). Extrinsic life goals and health㏑isk behaviors in adolescents. J. Appl. Soc. Psychol. 30, 1756–1771. doi: 10.1111/j.1559-1816.2000.tb02466.x

[ref45] Wu C. Palmer M. H. Sha K. (2020). Professional identity and its influencing factors of first-year post-associate degree baccalaureate nursing students: a cross-sectional study. Nurse Educ. Today 84:104227. doi: 10.1016/j.nedt.2019.104227, PMID: 31683135

[ref46] Yu H. Huang C. Chin Y. Shen Y. Chiang Y. Chang C.. (2021). The mediating effects of nursing professional commitment on the relationship between social support, resilience, and intention to stay among newly graduated male nurses: a cross-sectional questionnaire survey. Int. J. Environ. Res. Public Health 18:7546. doi: 10.3390/ijerph18147546, PMID: 34299995 PMC8307529

[ref47] Yu F. Raphael D. Mackay L. Smith M. King A. (2019). Personal and work-related factors associated with nurse resilience: a systematic review. Int. J. Nurs. Stud. 93, 129–140. doi: 10.1016/j.ijnurstu.2019.02.014, PMID: 30925279

[ref48] Yu X. Zhang J. Yu X. N. Zhang J. X. (2007). Factor analysis and psychometric evaluation of the connor-Davidson resilience scale (CD-RISC) with Chinese people. Soc. Behav. Pers. 35, 19–30. doi: 10.2224/sbp.2007.35.1.19

[ref49] Zeabadi S. M. Hasandoost F. Momeni M. Goudarzian A. H. Hosseinigolafshani S. (2021). Predictors of cognitive emotion regulation strategies: Iranian nurses. J. Educ. Health Promot. 10:188. doi: 10.4103/jehp.jehp_1002_20, PMID: 34250122 PMC8249965

[ref50] Zhang Z. Fu W. Tian C. Zhang F. Zhao B. Mao J.. (2021). Professional identity of Chinese nursing students during the COVID-19 pandemic outbreak: a nation-wide cross-sectional study. Nurse Educ. Pract. 52:103040. doi: 10.1016/j.nepr.2021.103040, PMID: 33813343 PMC9760126

[ref51] Zhang W. Liu Y. L. (2016). Demonstration of caring by males in clinical practice: a literature review. Int. J. Nurs. Sci. 3, 323–327. doi: 10.1016/j.ijnss.2016.07.006

[ref52] Zhao Z. H. Guo J. Y. Zhou J. Qiao J. Yue S. W. Ouyang Y. Q.. (2023). Perceived social support and professional identity in nursing students during the COVID-19 pandemic era: the mediating effects of self-efficacy and the moderating role of anxiety. BMC Med. Educ. 23:117. doi: 10.1186/s12909-022-03968-6, PMID: 36803504 PMC9936494

[ref53] Zhou Q. Yang X. Y. Wang Y. Feng Y. Luo S. (2021). Influencing factors of professional identity of undergraduate nursing male students in Sichuan Province. Chin. Occup. Med. 48, 293–296. doi: 10.11763/j.issn.2095-2619.2021.03.010

[ref54] Zimet G. D. Powell S. S. Farley G. K. Werkman S. Berkoff K. A. (1990). Psychometric characteristics of the multidimensional scale of perceived social support. J. Pers. Assess. 55, 610–617. doi: 10.1080/00223891.1990.9674095, PMID: 2280326

